# Iridoschisis bilatérale isolé

**DOI:** 10.11604/pamj.2016.24.210.3628

**Published:** 2016-07-08

**Authors:** Kriet Mohamed, Aitlhaj Lhousseine, Bouia Youssef, Bennouk Youssef

**Affiliations:** 1 Service d’Ophtalmologie, Hôpital Militaire Avicenne, Marrakech, Maroc

**Keywords:** Iridoschisis, atrophie irienne, athologie dégénérative, Iridoschisis, iris atrophy, degenerative disease

## Abstract

L’iridoschisis est une pathologie dégénérative rare au cours de laquelle le stroma irien antérieur se clive spontanément du feuillet postérieur. Le feuillet antérieur se dissocie alors en fibrilles flottant dans l’humeur aqueuse. Nous rapportons le cas d’une patiente de 70 ans, consulta pour une baisse d’acuité visuelle progressive et chez qui l’examen biomicroscopique a pu mettre évidence une cataracte sénile évolutive avec un iridoschisis bilatérale isolé. Les causes fréquentes d’atrophie irienne acquises (herpès, dispersion pigmentaire, traumatisme…) sont éliminées. Au cours du suivi, l’atrophie évolue avec multiplication des foyers d’atrophie de deux coté. Le tonus oculaire et le champ visuel restaient normaux. Le diagnostic de cette entité est important puisque son association fréquente au glaucome en impose son dépistage dans le même temps et suivi à long terme.

## Abstract (en)

Iridoschisis is a rare degenerative disease in which the anterior iris stroma spontaneously cleaves from the posterior layer. The anterior leaflet then disintegrates into fibrils floating in the aqueous humor We report the case of a 70 year-old woman who presented with progressive visual acuity loss. Biomicroscopic examination showed a progressive senile cataract with isolated bilateral iridoschisis. The common causes of acquired iris atrophy (herpes, pigment dispersion, trauma …) were eliminated. During follow-up, atrophy evolved by the multiplication of atrophy of both sides. Ocular tonicity and the visual field remained within the normal range The diagnosis of this entity is important since its frequent association with glaucoma imposes its detection and long-term monitoring.

## Introduction

L'iridoschisis est une pathologie dégénérative rare, bilatérale du sujet âgé se définit par un clivage entre le stroma irien et l'épithélium pigmenté dont le feuillet antérieur se fend en fibrilles et flotte librement dans l'humeur aqueuse. La présentation clinique la plus courante de l'iridoschisis sénile est une atrophie iris liée à l'âge associée à un glaucome. Nous rapportons le cas d'un iridoschisis bilatérale isolé.

## Patient et observation

**Figure 1:**
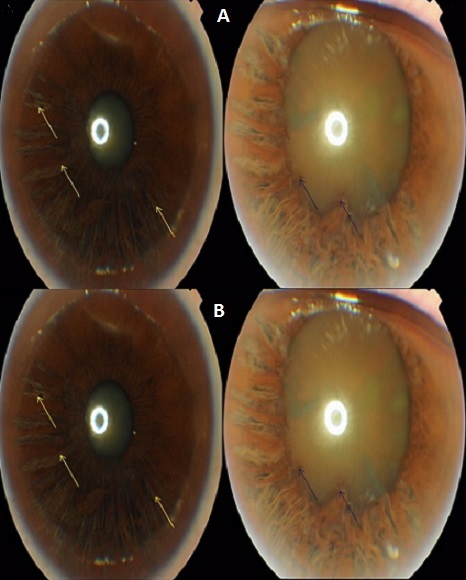
(A et B) trophie irienne touchant la moitié nasale de la circonférence irienne (flèche noire) OD avant et après dilatation, le feuillet antérieur se fend et flotte librement dans l’humeur aqueuse (flèche bleu)

Une femme âgée de 70 ans, antécédent d'asthme, consulta pour une baisse progressive d'acuité visuelle œil gauche. L'examen objectivait une acuité visuelle OD 8/10 P4 OG 4/10 P8. La pression intraoculaire par applanation était de 16 mm Hg à droit et de 15mm Hg à gauche. L'examen biomicroscopique mis en évidence une cornée transparente, une chambre antérieure de profondeur normale et atrophie irienne bilatérale symétrique touchant la moitié nasale de la circonférence irienne (Figure 1), les fibres du stroma irien étaient rompu depuis de la racine irienne jusqu'au sphincter et flottent librement dans l'humeur aqueuse. La patiente présentait une cataracte corticonucléiare bilatérale plus évoluée à l'œil gauche. La gonioscopie montrait un angle étroit mais sans synéchies antérieures. L'examen du FO montrait une papille de coloration normale et dont l'excavation ronde et symétrique est de 0.3. L'étude du champ visuel ne montrait pas de déficit glaucomateux. Le diagnostic d'un iridoschisis fut posé chez cette patiente sur l'aspect d'atrophie irienne bilatérale, l'âge avancé du patiente, l'absence de notion de traumatisme ou d'uvéite herpétique, de la transparence cornéenne et l'absence de déformation pupillaire. Lors du suivie, aucune déformation pupillaire n'est mise en évidence, l'atrophie est lentement évolutive avec multiplication des foyers d'atrophie de deux coté. Le tonus oculaire et le champ visuel restaient normaux.

## Discussion

L'iridoschisis est une pathologie dégénérative rare, bilatérale du sujet âgé survenant au cours de la sixième et septième décade. Au cours de laquelle une partie du stroma irien se sépare en deux feuillets dont la portion antérieure se fend en fibrilles [1]. Elle aboutit à la destruction tardive et progressive du stroma irien antérieur. Très peu de cas d'iridoschisis ont été rapportés dans la littérature à ce jour [1, 2]. Biomicroscopiquement, la partie inférieure de l'iris est la plus fréquemment affectée. La surface antérieure se clive en filaments qui flottent dans la chambre antérieure, sans déformation pupillaire et la cornée est habituellement claire [3]. L'étiopathogénie est encore mal élucidée.

Plusieurs hypothèses sont discutées: atrophie vasculaire ischémique, dégénérescence secondaire (traumatisme, synéchies postinflammatoires), ou encore prédisposition congénital [4]. Loewenstein et Foster ont évoqué, l'hypothèse de l'existence d'une substance lytique présente dans l'humeur aqueuse qui, dans des conditions anatomiques prédisposées, provoqueraient un clivage de l'iris [5]. Par contre Albers et Klein ont attribué l'iridoschisis comme la sclérose des vaisseaux sanguins augmente dans la partie antérieure du stroma, l'action de constriction et dilatation de l'iris provoqueraient ainsi une dissociation de la face antérieure du stroma irien [6].

L'étude histopathologique retrouve un tissu irien fibrosé et atrophique avec des couches pigmentées du stroma postérieur disposées de manière irrégulière avec un amincissement du stroma irien avec une diminution du nombre de fibres de collagènes alors que l'aspect de nerfs et vaisseaux sanguins sont normaux dans cette zone [1, 3].

En angiographie irienne, on peut observer des réductions de calibre vasculaire et des diffusions de fluorescéine en zone d'atrophie [7]. Le diagnostic positif de l'iridoschisis est clinique reposant sur l'aspect biomicroscopique du stroma irien qui se sépare en deux couches, un feuillet antérieur qui se fend en fibrilles qui fluctuent dans l'humeur aqueuse et un feuillet postérieur qui reste attaché au muscle dilatateur et à l'épithélium pigmenté rétinien.

Le diagnostic différentiel se pose essentiellement avec le syndrome irido-cornéo-endothélial (ICE syndrome) et le syndrome de dispersion pigmentaire en l'absence de faisceau de Krükenberg et la pigmentation normale du trabéculum, les séquelles de crise de fermeture d'angle, d'uvéite herpétique ou encore post-traumatiques [1, 8]. Chez l'adulte, L'iridoschisis est le plus souvent bilatérale asymétrique, isolé comme l'illustre notre observation. Elle survient généralement après 50 ans, avec un sex-ratio équilibré.

Un glaucome est fréquemment associé, approximativement, il s'agit d'un glaucome par fermeture d'angle dans près de 40% des cas [9, 10]. En général, le glaucome par fermeture de l'angle résulte de l'apposition de l'iris périphérique au trabéculum avec formation de synéchies antérieures périphériques et obstruction de l'évacuation de l'humeur aqueuse [8, 11]. Des associations ont été, également, rapportées avec subluxation du cristallin [12], Kératite interstitielle syphilitique [13], d'iris plateau, kératocône [4, 5], d'iris plateau [14].

## Conclusion

L'iridoschisis est pathologie dégénérative irienne acquise rare, habituellement bilatérale caractérisée par une dégénérescence du feuillet stroma antérieur de l'iris et son association fréquente de glaucome secondaire. Le glaucome n'était pas une caractéristique chez cette patiente, mais comme 65% des patients atteints d'iridoschisis présentaient un glaucome, il est possible que cette patiente pourrait développer un glaucome à des années plus tard.
